# Antiplasmodial and Antipyretic Activity and Safety Evaluation of the Methanolic Leaf Extract of *Murraya exotica (L.)*

**DOI:** 10.1155/2020/1308541

**Published:** 2020-08-05

**Authors:** Arnold Donkor Forkuo, Kwesi Boadu Mensah, Elvis Ofori Ameyaw, Aaron Opoku Antwi, Nana Kofi Kusi-Boadum, Charles Ansah

**Affiliations:** ^1^Department of Pharmacology, Faculty of Pharmacy and Pharmaceutical Sciences, Kwame Nkrumah University of Science and Technology, Kumasi, Ghana; ^2^Department of Biomedical and Forensic Sciences, School of Biological Science, College of Agriculture and Natural Sciences, University of Cape Coast, Cape Coast, Ghana

## Abstract

**Background:**

The increasing mortality and morbidity of malaria in Africa coupled with the recent reports of antimalarial drug resistance reinforces the need for novel antimalarial agents from natural plant products with folkloric use for the disease. *Murraya exotica (L.)* (Rutaceae) is widely used as an ornamental plant used indigenously to treat fever, cough, and infectious wounds and eliminate pain from injury and trauma. This study was conducted to evaluate extracts of the leaves of *Murraya exotica (L.)* (Rutaceae) for its safety and antipyretic and antimalarial activity in rodent models.

**Method:**

In this study, the Peters 4-day suppressive and curative test in *Plasmodium berghei*-infected mice was used to demonstrate the antiplasmodial activity of the methanolic leaf extract of *Murraya exotica (L.)* (MEE). The study also evaluated the subacute toxicity study and the antipyretic activity of MEE on baker's yeast-induced hyperthermia in rodent models.

**Results:**

*Murraya exotica (L.)* extract demonstrated curative antimalarial activity, with a percentage suppression of 45.84, 64.32 ± 0.33, 56.74 ± 2.16, and 64.61 ± 0.67 at doses of 50, 100, 300, and 600 mg/kg, respectively. In the Peters 4-day suppressive test, MEE at dose 600 mg/kg had the highest chemosuppression (76.02 ± 1.38%) compared with artesunate (2 mg/kg, *p.o.*) (82.56 ± 0.97%). Subacute oral toxicity studies in Sprague-Dawley rats documented no deaths, with no significant changes in clinical signs, organ weights, and hematological and biochemical parameters. The LD_50_ of MEE was estimated to be above 1000 mg/kg in Sprague-Dawley rats. All doses of MEE and paracetamol reduced pyrexia in 1 h and 2 h after their administration. The percentage reduction of rectal temperature (*T*_*R*_) for the positive control (paracetamol, 150 mg/kg, *p.o.*) was 44.36% while the *Murraya exotica* extract at doses 50 mg/kg, 100 mg/kg, 300 mg/kg, and 600 mg/kg recorded 67.74%, 40.78%, 66.42%, and 59.42%, respectively. *Murraya exotica* at dose 100 mg/kg exhibited significant reduction (*p* < 0.05) in baker's yeast-induced pyrexia.

**Conclusions:**

The findings in this study show the antipyretic, curative, and suppressive antiplasmodial activity as well as the safety of the methanolic leaf extract of *Murraya exotica (L.)* supporting its traditional use for malaria and fever.

## 1. Background

Despite several years of intense research, malaria remains a deadly worldwide disease. According to the World Health Organization [1], about 219 million cases of malaria were reported in 90 countries with 435,000 deaths in 2017. The WHO African Region was home to 92% of these malaria cases and 93% of malaria deaths. Antimalarial drug resistance remains a major hurdle in the global effort to eradicate malaria [2]. The persistence of this global health problem is partly attributed to the development of resistance by the limited available antimalarials. The artemisinins, though effective in the global fight against malaria, are hampered by limited supply and high cost.

While there is much need for more antimalarial agents, the drug development pipeline remains woefully thin, with little chemical diversity. Currently, no clinically tested alternative to the valuable artemisinins has been developed [3]. Although vaccine development could be the surest long-term control option, clinical trials are still ongoing [4].

Plant-derived compounds have played a crucial role in the discovery and development of new drug molecules for the treatment of several diseases. Medicinal plants have been used for the prevention and treatment of malaria, and the isolation of new bioactive compounds from these plants offers novel, affordable, and efficient options that could serve as primary molecules for antimalarial treatment with artemisinin and quinine being classical examples [5].

*Murraya exotica (L.)* (Rutaceae) is an example of a medicinal plant that has been used traditionally in the treatment of malaria [6] with no scientific credence. In southern China where the plant is actively grown, *Murraya exotica (L.)* (Rutaceae) has been well documented in the Pharmacopoeia of the People's Republic of China for the treatment of rheumatic arthralgia, stomachache, fever, body swelling, toothache, and pain [7]. Wu et al. [8] have demonstrated the antinociceptive and anti-inflammatory activities of 70% ethanol extracts of *M. exotica* in rat knee osteoarthritis models.

This study is aimed at evaluating the *in vivo* antiplasmodial, safety, and antipyretic properties of the methanolic leaf extract of *Murraya exotica (L.)* (Rutaceae) in rodent models.

## 2. Methods

### 2.1. Plant Collection and Authentication

The fresh leaves of *Murraya exotica (L.)* were collected in Bekwai, Ashanti region (August 2017). Dr. George Henry Sam of the Department of Herbal Medicine identified and authenticated the plant using organoleptic analysis. A voucher specimen (KNUST/H/M/2017/M007) of the leaves of *Murraya exotica (L.)* was kept at the herbarium of the Faculty of Pharmacy and Pharmaceutical Sciences, Kwame Nkrumah University of Science and Technology (KNUST), Kumasi, Ghana.

### 2.2. Extraction of Plant Material

The fresh leaves were washed and air-dried for 2 weeks under shade. The dry leaves obtained were then milled into powder with a laboratory scale mill. The powdered leaves (2 kg) were extracted by maceration with 10 l of 70% methanol for 72 hours at room temperature and then concentrated under reduced pressure at 40°C into an oily mass in a rotary evaporator. The extract was further dried in a hot air oven at 40°C for 3 days and then kept in a refrigerator for later use. The final yield was 24.10% (*w*/*w*). The crude extract obtained is subsequently referred to as *Murraya exotica* extract (MEE) or the extract in this study. The various concentrations of the methanolic extract were prepared in 5% sodium carboxymethylcellulose solution for the experimental procedures.

### 2.3. Experimental Animals

Male Sprague-Dawley rats (125–167 g) and ICR-strain mice (18–22 g) between 6 and 8 weeks were used. They were obtained from Noguchi Memorial Institute for Medical Research (NMIR), University of Ghana, Legon, and kept in the animal house of the Department of Pharmacology, KNUST. The animals were housed in stainless steel cages and maintained under normal animal housing conditions. This involved the monitoring of room conditions, monitoring of animals for health problems and pregnancy, proper cage enclosure conditions, food and water levels, proper ventilation, light, temperature, and sanitation. Rats and mice were fed a commercial pellet diet and granted access to clean water.

### 2.4. Rodent Parasite

The species of malaria parasite used to infect the mice was *Plasmodium berghei* NK 65 and was obtained from Noguchi Memorial Institute for Medical Research. The parasites were kept alive by intraperitoneal passage in mice after ≥5% parasitemia has been established.

### 2.5. Chemicals and Reagents

Methanol, ethanol, ferric chloride (FeCl_3_), hydrochloric acid (HCl), Dragendorff's reagent, sulphuric acid (H_2_SO_4_), sodium hydroxide (NaOH), chloroform, acetic anhydride, sodium carboxymethylcellulose, 10% formalin, and 10% Giemsa. All the chemicals used were of analytical grade.

### 2.6. Phytochemical Screening of *Murraya exotica* Extract

Standard laboratory methods described by Vaghasiya et al. [9] were used in the phytochemical screening of secondary metabolites of the methanol extract of the leaves of *Murraya exotica* (MEE).

### 2.7. Peters 4-Day Suppressive Test

The *in vivo* antimalarial activity of *Murraya exotica* extract (MEE) was assessed using the 4-day suppressive test in the *P. berghei*-infected mouse model [10]. Mice infected with the *P. berghei* NK 65 strain served as the reservoir, parasites were maintained by serial blood passage in mice, and the blood stage was stored at −80°C until use. The donor mice were infected with 200 *μ*l of *P. berghei* parasite inoculum. The parasitized blood of each donor mouse was collected from the tail vein and diluted with 0.9% sodium chloride. ICR mice of both sexes were divided into five groups and each intraperitoneally infected with 0.2 ml of saline suspension containing 1.0 × 10^7^ parasitized erythrocytes (day 0). Three hours after infection, the mice in each group (*n* = 6) were treated with oral daily doses of 50, 100, 300, or 600 mg/kg body weight of MEE for four consecutive days (test groups 1, 2, 3, and 4, respectively). Positive and negative control groups were treated daily with an oral daily dose of artesunate at 2 mg/kg body weight and 5% sodium carboxymethylcellulose, respectively. The parasitemia of each mouse was determined under light microscope by examination of Giemsa-stained thin blood smears prepared from the mouse tail 4 days (96 hours) post infection [11].

### 2.8. Antiplasmodial Curative Test

In this study, the antiplasmodial curative activity of the methanolic extract of *Murraya exotica* was investigated using the Ryley and Peters method [12]. Thirty-five (35) mice (18-25 g) were inoculated intraperitoneally with 1.0 × 10^7^ cells/mm^3^ of *Plasmodium berghei* NK 65 parasite. Parasitemia was confirmed after 72 hours, and the mice were randomly divided into 6 groups of 5 mice per group. Groups 5 and 6 served as the positive and negative controls, respectively. The positive control group was treated with artesunate 2 mg/kg orally, and an equivalent volume of 5% sodium carboxymethylcellulose was given to the negative control group. Groups 1, 2, 3, and 4 were treated with 50 mg/kg, 100 mg/kg, 300 mg/kg, and 600 mg/kg of the plant extract, respectively. The treatment was daily for 5 days, and the oral route was used in each group. Blood samples were taken from the tail vein of each mouse onto a microscope slide to make thin films [13]. Blood smears were taken on days 1, 3, and 6 of drug treatment. The thin films were prepared by fixing the blood on the slide with methanol, then staining the slide with 10% Giemsa for 10 minutes. The thin films prepared were examined microscopically in order to establish the level of parasitemia.

The mean parasitemia in each group of mice for both the curative and the suppressive test was used to calculate the % suppression for each dose using the following formula:

(1) $$\begin{array}{c} \%\text{parasitemia}=\frac{\text{Number of parasitized RBCs}}{\text{Total number of RBCs counted}}\times 100. \end{array}$$

Average percentage chemosuppression was calculated as

(2) $$\begin{array}{c} \%\text{suppression}=\frac{\%\text{parasitemia in the negative control}-\%\text{parasitemia in the test }}{\%\text{parasitemia in the negative control}}\times 100. \end{array}$$

### 2.9. Antipyretic Test

The effect of drugs on baker's yeast-induced hyperthermia as described by Tomazetti et al. [14] and Boakye-Gyasi et al. [15] was employed. A 2-day habituation session was conducted where rectal temperatures (*T*_*R*_) of the rats were recorded by inserting a lubricated digital thermometer (external diameter: 3 mm, 0.1°C precision) 3 cm into the rectum of rats. Rats with initial rectal temperature (*T*_*R*_) between 36 and 37°C were selected for these antipyretic tests. A pyogenic dose of baker's yeast (0.135 g/kg, *i.p.*) was injected in each animal on the third day after measuring basal temperatures. Changes in rectal temperature (*T*_*R*_) were recorded every hour up to 4 h. Rats with a rise of not less than 0.5°C in rectal temperature were selected for the experiment. Animals were randomly divided into six groups of five rats each. Group 1 received paracetamol (150 mg/kg, *p.o.*). Groups 2, 3, 4, and 5 received MEE 50, 100, 300, and 600 mg/kg, *p.o.*, respectively. Group 6 did not receive any drug/extract after the yeast administration. Another group of 5 rats (Group 7) received only normal saline (0.9% NaCl, *i.p.*) without baker's yeast administration. *T*_*R*_ were monitored hourly over the following 4 h period after extract/drug administration.

### 2.10. Subacute Toxicity Test

The oral subacute toxicity study of *Murraya exotica* methanolic leaf extract was carried out in Sprague-Dawley rats using the modified Locke test [16]. Sprague-Dawley rats, weighing 125–167 g were placed in 5 treatment groups. The negative control group (group 5) received normal saline. Various test groups of *Murraya exotica* were groups 1, 2, 3, and 4 which received doses of 100, 250, 500, and 1000 mg/kg, *p.o.*, respectively. The rats were observed for weakness, stimulation, anorexia, sleep, coma, and death in the first five hours and subsequently for 14 days. The variations in weights on day 1, day 7, and day 14 were as well investigated by taking the animals' weights on a balance on those days. On day 15, the rats were sacrificed by cervical dislocation, the jugular vein cut, and blood allowed to flow freely into tubes with and without ethylenediaminetetraacetic acid (EDTA) as coagulant. They were dissected, and their organs (lungs, liver, heart, and spleen) were weighed individually. The organs were preserved thereafter in 10% formalin. The blood samples of the animals were as well collected in EDTA and plain tubes for hematological and biochemical analyses, respectively.

### 2.11. Hematological Parameters

Twenty-four hours after the last dose, the animals were sacrificed by cervical dislocation and the blood samples were collected by cardiac puncture. The blood samples for hematological parameters (white blood cell count, red blood cell count, hemoglobin, platelet count, and packed cell volume) were collected into EDTA containers and analyzed using an automated machine (Automated CBC Analyzer: Sysmex KX-21).

### 2.12. Biochemical Analysis

The blood samples for biochemical parameters (globulin, albumin, alkaline phosphatase, indirect bilirubin, direct bilirubin, total bilirubin, aspartate transaminase, alanine transaminase, gamma-glutamyl transpeptidase, urea, and creatinine) were collected into EDTA tubes and analyzed using an automated analyzer (automated biochemical analyzer).

### 2.13. Ethical Consideration

The use and handling of animals were in agreement with the National Institutes of Health Guidelines for Care and Use of Laboratory Animals (1985) and was approved by the Institutional Ethical Review committee of the Department of Pharmacology, Kwame Nkrumah University of Science and Technology (KNUST) (No. PHARM/ETHIC/ET194/19).

### 2.14. Statistical Evaluation

The statistical analysis of data obtained was analyzed using GraphPad V6.0 (GraphPad Prism software, San Diego, USA). The treatment groups and the controls were analyzed and compared using the one-way analysis of variance (ANOVA). The results obtained were expressed as mean ± SEM. The antimalarial activity of MEE was determined from the ratio of percentage of parasite reduction in treated and negative control groups.

## 3. Results

### 3.1. Phytochemical Screening

The preliminary phytochemical screening of the dried methanolic leaf extract of *Murraya exotica* showed the presence of tannins, saponins, coumarins, alkaloids, flavonoids, glycosides, and sterols.  Table 1 shows the results of the phytochemical screening.

### 3.2. Antiplasmodial Studies

#### 3.2.1. Rane/Curative Test

ICR mice were treated with 50 mg/kg, 100 mg/kg, 300 mg/kg, and 600 mg/kg of *Murraya exotica* extract and 2 mg/kg of artesunate (positive control). After day 3 and day 6 of treatment, the positive control (artesunate 2 mg/kg) showed the highest percentage suppression of 57.02 ± 0.33 and 89.13 ± 0.0, respectively. *Murraya exotica* also showed a significant decrease in the parasitemia after day 3 which further decreased on the 6^th^ day. The highest dose of *Murraya exotica* extract administered (600 mg/kg) showed the highest parasitemia suppression (64.61%).  Table 2 shows the results obtained from the antimalarial study.

### 3.3. Peters 4-Day Suppressive Test

*In vivo* 4-day suppressive assay results for the leaf extract of *Murraya exotica* using *Plasmodium berghei*-infected mice are summarized in Table 3. 96 hour postinfection, MEE at doses of 50 mg/kg, 100 mg/kg, 300 mg/kg, and 600 mg/kg showed a percentage suppression of 61.55 ± 1.87, 69.20 ± 1.73, 72.42 ± 1.55, and 76.02 ± 1.38, respectively. The positive control (artesunate, 2 mg/kg) assayed in parallel reduced parasitemia by 82.56 ± 0.97% with no observed mortality in the group after 30 days.

### 3.4. Antipyretic Activity

**Figure 1:**
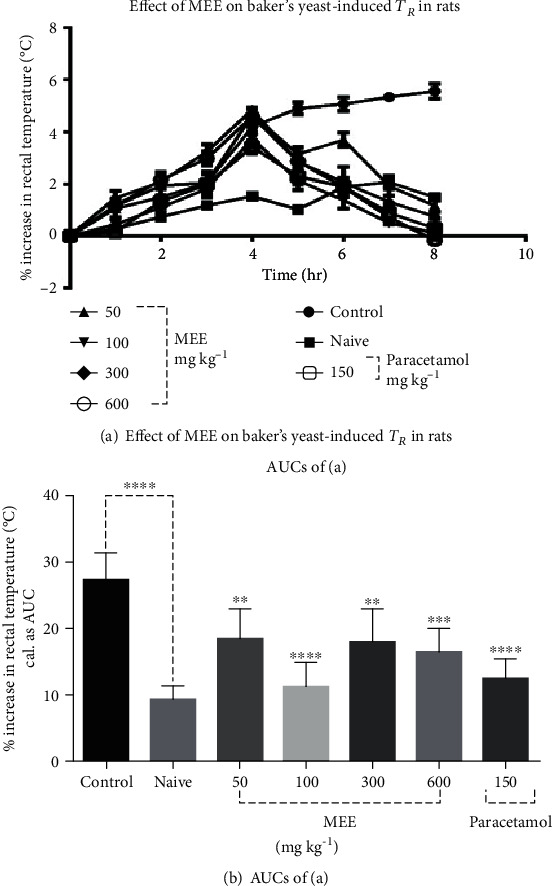
Effect of MEE 50-600 mg/kg, *p.o.*, and paracetamol (150 mg/kg, *p.o.*) on time-course curve (a) and the total increase in temperature (calculated as AUCs (b)) on baker's yeast-induced changes of rectal temperatures in rats. Naive represents animals with no treatment with yeast. Values are expressed as mean ± SEM (*n* = 5). ^∗∗∗∗^*p* < 0.001 compared to vehicle-treated group (control) (ordinary one-way ANOVA comparison test with descriptive statistics).

MEE at all doses reduced pyrexia in the Sprague-Dawley rats. The inhibition remained significant up to 4 h of administration. MEE at 100 mg/kg dose showed the maximum antipyretic effect and returned the body temperature to normal levels (*p* > 0.05), almost as effective as the standard drug paracetamol (Figure 1).

### 3.5. Subacute Toxicity

The subacute toxicity study revealed that the methanolic extract of *Murraya exotica* was safe up to 1000 mg/kg.

### 3.6. Relative Organ Weight

With regard to the relative organ weight, it was observed that the target organs, heart, kidney, liver, and spleen, of the Sprague-Dawley rats in the test groups did not differ significantly from those of the control group as shown in Table 4.

### 3.7. Hematological Analysis

In the hematological screening, the following blood parameters assessed, red blood cell count, platelet count, white blood cell count, hemoglobin level, and cell volume, revealed that the methanolic extract of *Murraya exotica* at doses 100 mg/kg, 250 mg/kg, 500 mg/kg, and 1000 mg/kg did not produce any significant effects when compared to the control.  Table 5 shows the results obtained from the hematological analysis.

### 3.8. Biochemical Analysis

In the biochemical analysis, the various parameters assessed revealed that the methanolic extract of *Murraya exotica* at doses 100 mg/kg, 250 mg/kg, 500 mg/kg, and 100 mg/kg did not produce any significant effects on the test groups in comparison to the control group.  Table 6 shows the results obtained from the biochemical analysis.

## 4. Discussion

The development of resistance to the commonly used antimalarial drugs and its resultant increase in morbidity and mortality due to malaria continue to pose a major public health threat [17]. To address this global threat caused by *Plasmodium falciparum* malaria, novel antimalarial drugs and potent vaccines are urgently needed. *Murraya exotica* grows widely in southern Asia, and it is used as an ornamental and hedge plant for its pleasant smell and beauty. However, the leaves and roots of the plant have been traditionally used as medicine to treat rheumatalgia, toothache, malaria, and body pains from injury and trauma [6, 18]. In this study, the *in vivo* antiplasmodial and antipyretic activity and the safety profile of the methanolic leaf extract of the plant were assessed.

Phytochemical analysis showed that the *Murraya exotica* extract contains tannins, saponins, coumarins, alkaloids, flavonoids, glycosides, and sterols. Other studies performed also show that *Murraya exotica* extracts contain carbohydrates, proteins, amino acids, and phenolic compounds [19], phytosterol and coumarins [20], alkaloids and flavonoids [21], and flavone derivatives [22]. These compounds may be responsible for the biological activity of the plant. A compound isolated from *Murraya exotica*, umbelliferone, was reported to have antihyperlipidemic and antidiabetic activities [23]. The similarity in the phytochemicals reported with earlier studies confirms the identity of the plant used in the biological assays.

Subacute toxicity studies are important to establish the safe use of plant products for human use. Analyses of hematological and biochemical parameters, and relative organ weight present an effective measure of assessing the safety profile of a drug or plant product. The 14-day subacute toxicity study in rat showed no signs of toxicity, behavioral changes, and mortality when methanolic extracts of *Murraya exotica* up to 1000 mg/kg were administered compared to the control groups.

In toxicology studies, comparison of organ weights between treated and untreated groups of animals is used conventionally to evaluate the toxic effect of the test substance [24]. The usefulness of relative organ weight in toxicity studies includes sensitivity to predict toxicity, enzyme induction, physiologic perturbations, and acute injury; it correlates well with histopathological changes, and there is little interanimal variability [25]. There were no significant differences in the weights of the target organs of the treatment groups when compared to the control group (Table 4). This indicates that the methanolic extract *Murraya exotica* treatment for 14 days did not cause any detrimental effect on the heart, kidney, liver, and spleen of the Sprague-Dawley rats in the various treatment groups.

Most toxic compounds target the hematopoietic system, an important index of physiology, and hence help to determine the pathological status in man and animals [26]. In the hematological analysis, the values of the different parameters assessed [red blood cell (RBC), hemoglobin (Hb), hematocrit (HCT), white blood cell (WBC), mean cell hemoglobin (MCH), mean cell hemoglobin concentration (MCHC), and platelet] for the treated groups were within normal biological and laboratory limits when compared with the values of the control group. No significant changes were observed in all the treated groups as compared to the control groups. This indicates that the *Murraya exotica* extract does not show significant adverse effects on the blood, blood cells, and target organs at the doses used. In the biochemical assays, there was no significant increase in the levels of the parameters measured at different doses (Table 6) in the different groups of animals treated with the extract as compared to the control. *Murraya exotica* extract at different dose levels (up to 1000 mg/kg) tested did not produce considerable change in the levels of the different parameters measured accounting for the safety of the plant in human use.

The *in vivo* curative antiplasmodial study showed that the plant extract demonstrated antimalarial activity with the highest percentage of parasitemia suppression exerted by group 4 (64.61), that is, mice receiving the highest dose of 600 mg/kg of the *Murraya exotica* extract. The positive control group receiving artesunate 2 mg/kg exerted 89% suppression. The rise in percentage suppression observed in the various groups was not in proportion to the dose administered. Group 2 (100 mg/kg MEE) for instance recorded a percentage suppression of 33.67 on day 3 and 64.32 on day 6 whereas group 3 (300 mg/kg MEE) with a higher dose recorded a percentage suppression of 19.91 and 56.74 on days 3 and 6, respectively. In the 4-day suppressive antiplasmodial assay, MEE produced a dose-dependent chemosuppression, with the lowest dose tested (50 mg/kg) producing significant reduction in parasitemia (61.55%). The highest dose of MEE tested (600 mg/kg) in group 4 had a percentage suppression of 76.02% and did not achieve the level of chemosuppression seen with the positive control—artesunate (2 mg/kg) which had a suppression of 82.56% on the 5^th^ day. The results indicate that the methanolic leaf extract of *Murraya exotica* possesses both suppressive and curative antiplasmodial activity at the doses tested.

MEE at all doses (50, 100, 300, and 600 mg/kg) reduced the baker's yeast-induced pyrexia in the Sprague-Dawley rats. These inhibitions remained significant up to 4 h of administration. MEE at 100 mg/kg showed the maximum antipyretic effect and returned body temperature to normal levels (*p* > 0.05), almost as effective as the standard drug paracetamol at 150 mg/kg.

The antipyretic effects of MEE observed might be due to possible reduction in the brain concentration of prostaglandin E_2_ especially in the hypothalamus through its action on COX-3 or by the enhancement of the production of the body's own antipyretic substances like vasopressin and arginine by pharmacologically active metabolites. These antipyretic substances are known to reduce proinflammatory mediators, improve anti-inflammatory signals at sites of injury, or increase antipyretic messages within the brain [27].

A secondary plant metabolite such as alkaloids, quassinoids, and sesquiterpene lactones has been shown to possess antimalarial activity [28]. The phytochemical screening of the dried methanolic leaf extract of *Murraya exotica* showed the presence of tannins, saponins, coumarins, alkaloids, flavonoids, glycosides, and sterols. Although the active constituents in the plant extract responsible for the antimalarial effects are not known or have yet to be identified, the presence of alkaloids, glycosides, tannins, and flavonoids has been implicated in antipyretic and antiplasmodial activity and might be as a result of a single, additive, or synergistic action of these compounds [29, 30].

## 5. Conclusion

The findings in this study show that the methanolic extract of *Murraya exotica* is safe in Sprague-Dawley rats and demonstrate both suppressive and curative antiplasmodial activity in *Plasmodium berghei*-infected mice. MEE also possessed considerable antipyretic properties at the doses tested in the baker's yeast-induced pyrexia in rodent models. This study gives support to the claim for the traditional use of the plant in the treatment of malaria and fever.

## Figures and Tables

**Table 1:** Secondary metabolized in the leaves of *Murraya exotica (L.)*.

Secondary metabolite	Presence
Tannins	+
Saponins	+
Coumarins	+
Alkaloids	+
Flavonoids	+
Glycosides	+
Sterols	+

Key: +: represents present.

**Table 2:** Percentage suppression of *Plasmodium berghei* by four dose points of *Murraya exotica* and artesunate (2 mg/kg) on day 3 and day 6, respectively.

Treatment	Day 3 (%)	Day 6 (%)
Group 1 (50 mg/kg)	20.60 ± 0.33	45.84 ± 0.00
Group 2 (100 mg/kg)	33.67 ± 2.23	64.32 ± 0.33
Group 3 (300 mg/kg)	19.91 ± 0.67	56.74 ± 2.16
Group 4 (600 mg/kg)	26.19 ± 0.00	64.61 ± 0.67
Positive control (2 mg/kg)	57.02 ± 0.33	89.13 ± 0.00

Values expressed as means ± SEM (*n* = 5), compared to the control by Dunnett's multiple comparison test.

**Table 3:** Percentage suppression of *Plasmodium berghei* by four dose points of *Murraya exotica* and artesunate (2 mg/kg) on day 4.

Treatment	% suppression
Group 1 (50 mg/kg)	61.55 ± 1.87
Group 2 (100 mg/kg)	69.20 ± 1.73
Group 3 (300 mg/kg)	72.42 ± 1.55
Group 4 (600 mg/kg)	76.02 ± 1.38
Positive control (2 mg/kg)	82.56 ± 0.97

Values expressed as means ± SEM (*n* = 5), compared to the control by Dunnett's multiple comparison test.

**Table 4:** Effect of the methanolic extract of *Murraya exotica* on organ weights of rats treated for 14 days.

Organ			Relative organ weight		
	Control	100 mg/kg	250 mg/kg	500 mg/kg	1000 mg/kg
Liver	2.87 ± 0.04	3.40 ± 0.30	3.03 ± 0.14	3.33 ± 0.19	2.94 ± 0.08
Kidneys	0.50 ± 0.04	0.61 ± 0.04	0.62 ± 0.02	0.64 ± 0.02	0.58 ± 0.06
Spleen	0.38 ± 0.08	0.47 ± 0.03	0.46 ± 0.08	0.50 ± 0.10	0.45 ± 0.02
Heart	0.41 ± 0.07	0.37 ± 0.03	0.39 ± 0.01	0.40 ± 0.02	0.41 ± 0.02

Values are expressed as mean ± SEM (*n* = 5) compared to the control by the Newman-Keuls test.

**Table 5:** Effects of the methanolic extract of *Murraya exotica* on the hematological parameters of Sprague-Dawley rats treated with the aqueous extract for 14 days.

Parameters	Control	100 mg/kg	250 mg/kg	500 mg/kg	1000 mg/kg
RBC	8.06 ± 0.44	7.43 ± 0.7	7.38 ± 0.17	7.37 ± 0.1	8.16 ± 0.67
HGB	14.13 ± 0.57	13.43 ± 0.53	13.5 ± 0.46	12.9 ± 0.4	13.87 ± 0.74
HCT	52.97 ± 3.6	49.87 ± 2.12	49.13 ± 1.84	48.43 ± 1.13	49.9 ± 2.7
MCV	65.6 ± 1.35	67.8 ± 3.67	66.6 ± 2.13	65.7 ± 0.3	61.43 ± 1.89
MCH	17.6 ± 0.32	18.27 ± 1.01	18.3 ± 0.5	17.5 ± 0.1	17.1 ± 0.78
MCHC	26.8 ± 1.01	26.93 ± 0.09	27.47 ± 0.12	26.6 ± 0.21	27.8 ± 0.5
PLT	507 ± 162.7	754.33 ± 52.3	821 ± 79.04	910.33 ± 170.04	768.33 ± 6.55
WBC	11.37 ± 2.72	11.13 ± 2.28	7.67 ± 0.64	9.23 ± 0.68	9.47 ± 0.62
LYM	75.1 ± 7.03	80.27 ± 5.09	73.17 ± 3.9	72.3 ± 6.07	61.33 ± 7.41
NEUT	24.9 ± 7.03	25.27 ± 5.10	30.25 ± 2.65	31.7 ± 6.45	41.65 ± 9.59

Values expressed as means ± SEM (n =3), compared to the control by Dunnett's multiple comparisons test.

**Table 6:** Effects of the methanolic extract of *Murraya exotica* on the biochemical parameters of Sprague-Dawley rats after 14 days of treatment.

Parameters	Control	100 mg/kg	250 mg/kg	500 mg/kg	1000 mg/kg
ALB	36.33 ± 0.88	36.33 ± 1.20	36.67 ± 1.86	37.00 ± 0.58	35.67 ± 0.33
GLB	45 + 1.0	46.00 ± 0.58	49.67 ± 3.35	48.33 ± 0.67	47.00 ± 2.52
T.PRO	81.33 ± 1.76	82.33 ± 0.88	79.67 ± 3.33	85.33 ± 0.33	82.67 ± 2.67
ALP	719 ± 42.17	688.33 ± 103.65	796.7 ± 3.39	692.33 ± 60.4	797.00 ± 71.85
ALT	215.33 ± 13.44	216.33 ± 12.67	194.33 ± 22.82	231.17 ± 36.1	212.67 ± 17.05
AST	17.67 ± 11.72	23.67 ± 2.60	54.33 ± 10.93	56.00 ± 39.96	61.67 ± 40.68
D.BIL	1.67 ± 0.33	2.00 ± 0.00	1.82 ± 3.37	1.67 ± 0.33	2 ± 0
IND.BIL	1 ± 0	1.00 ± 0.00	1.02 ± 3.38	0.33 ± 0.33	0.33 ± 0.33
T.BIL	2.67 ± 0.33	3.00 ± 0.00	2.84 ± 3.36	2 ± 0	2.33 ± 0.33
GGT	0.33 ± 0.33	2.33 ± 4.33	0.33 ± 0.67	0.54 ± 0.33	0.67 ± 0.67

Values expressed as means ± SEM (*n* = 3), compared to the control by Dunnett's multiple comparison test. ALB: albumin; GLB: globulin; T.PRO: total protein; ALP: alkaline phosphatase; ALT: alanine transaminase; AST: aspartate transaminase; D.BIL: direct bilirubin; IND.BIL: indirect bilirubin; T.BIL: total bilirubin; GGT: gamma-glutamyl transferase.

## Data Availability

All data generated or analyzed during this study are included in this published article.
